# Microbes are life, the biological core of *One Health* and *Health in All Policies*

**DOI:** 10.1093/femsml/uqaf012

**Published:** 2025-06-28

**Authors:** Kenneth Timmis, Fernando Baquero, James K Timmis, Margaret Douglas

**Affiliations:** Institute of Microbiology, Technical University of Braunschweig, 38106 Braunschweig, Germany; Ramón y Cajal Institute for Health Research (IRYCIS), Department of Microbiology, Ramón y Cajal University Hospital, 28034 Madrid, Spain; Department of Political Science, University of Freiburg, 79085 Freiburg, Germany; Athena Institute for Research on Innovation and Communication in Health and Life Sciences , Vrije Universiteit Amsterdam, Amsterdam, 1081 HV, The Netherlands; Department of International Health, Care and Public Health Research Institute – CAPHRI, Maastricht University, 6211 LK Maastricht, The Netherlands; Public Health Scotland, Edinburgh, EH12 9EB, United Kingdom; University of Glasgow, Glasgow G12 8TB, United Kingdom

## Health in All Policies

The World Health Organization (WHO) is promoting the operative concept of “Health in All Policies” (HiAP; https://iris.who.int/bitstream/handle/10665/112636/9789241506908_eng.pdf?sequence=1). This concept recognizes that population health is not merely the consequence of health sector programmes and that it is significantly impacted by policies that guide actions beyond the health sector, including those concerning transport, housing and urban planning, the environment, education, agriculture, finance, taxation, and economic development. HiAP seeks to couple policy development in essentially all sectors of government to considerations of health and inequities in health, and thereby reduce and ultimately eliminate ‘silo’ policy deliberations that may fail to protect against potential adverse health consequences. Although HiAP is concerned with human health, human health is highly dependent on the health of the biosphere (One Health). The wide list of policies ‘beyond the health sector’ under the umbrella of HiAP implies that all anthropogenic activities can affect health. We argue here that one essential reason for these impacts is that all anthropogenic activities exert effects on the microbiosphere—the microbial world—and that such effects are ‘nodal’ in the sense that human, animal, and vegetal life is absolutely dependent on microbes.

### Microbes are life—life is health

Microbes were and are the source of all life (Bertrand et al. 2015, Knoll 2015). They constitute the necessary platform, the foundation, of any conceivable form of life, and have furnished all forms of life with their basic cellular functions (e.g. Moore et al. 2017, Gumerov et al. 2022). Microbes are essential primary producers, acquiring their energy from sunlight and materials from nonliving sources to make ‘life’ building blocks and structures, and transferring such energy and building blocks both to other microbes and especially higher forms of life (note that, although it is generally perceived that this process is in the main carried out by plants, it is plant chloroplasts, which are derived from bacteria and constitute plant cell endosymbionts, that are the key players in photosynthesis). Chloroplasts, mitochondria, and other cellular endosymbionts constitute fundamental organelles of eukaryotic cells playing vital roles in energy transformations and metabolism (McCutcheon 2016). Moreover, the microbiota of all organisms—plants and animals—provide a myriad of goods and services vital for their metabolism, functioning and well-being.

Even without including the huge number of microorganisms intimately associated with higher organisms, the total biomass (about 80 gigatons of carbon) of all tiny free-living microorganisms on Earth—the microbiosphere—is at least 50 times higher than the biomass of all animals on Earth, most of these being marine animals (Bar-On et al. 2018). Bacteria, together with protists, constitute about 70% of the total biomass of marine life. These numbers clearly reflect the overwhelming density and ubiquity of the microbial world, the base of the entire pyramid of life.

But it is not just biomass. The signature characteristic of the microbial world is its exceptional diversity, both phylogenetic (one estimate suggests that the microbiosphere may contain one trillion species of microbes; Locey and Lennon 2016) and metabolic (an astonishing range of metabolic pathways consisting of millions of individual reactions yielding directly and indirectly a vast array of biochemicals, many thus far poorly characterized: the so-called ‘metabolic dark matter’; MohammadiPeyhani et al. 2022). This, coupled with their multitude of environmental monitoring-regulatory-response functions (e.g. Matilla et al. 2023a, b), assures a huge range of ecophysiologies enabling the life conquest and colonization of the more heterogeneous micro-niches on Earth, even those characterized by extreme conditions of heat, cold, acidity, radiation, osmotic pressure, or isolation (life inside rocks or in the upper atmosphere). Crucially, what constitutes the warp of life is the number, diversity, and responsiveness of connective interactions in the life network, many of which constitute vital—life-giving—ecosystem services. In this respect, microorganisms have an astronomic number of interactions, both within the microbiosphere, and with all other members of the biosphere. Neurobiologists talk about the ‘connectome’ in regard to the topology of neural connections in the brain (e.g. Zhang et al. 2024), but the microbiosphere constitutes a global biosphere connectome. Such a comprehensive level of dynamic connectivity, exponentially stimulated by noncellular biological linkers such as viruses and mobile genetic elements, determines the functioning, evolutionary development, health, stability, and resilience of the biosphere, the entire structure of life. Life on planet Earth might justifiably be considered as one metaorganism, with microbes as its source, principal actors, and matrix. The study of microbes has yielded much of our understanding of life processes, and how these can malfunction and result in disease. Microbes are life, and life is health.

## Microbes repair, regenerate, and autoregulate the functioning of the biosphere and its interfaces with the geosphere and atmosphere

The diversity of global microbial metabolism assures the cycling of elements and matter that make up the biosphere (Falkowski et al. 2008), including both biological and many nonbiological (e.g. industrial) wastes. Microbial element and matter cycling, and microbial responsiveness to chemical and energetic opportunities and environmental changes, endow them with extraordinary regenerative capacities that influence the health of the biosphere and all organisms in it.

Biosphere structure and function are, however, challenged by changes in the physical and chemical conditions of Earth, ranging from alterations in the structure of the planet by naturally occurring, often unpredictable, events (seismic events, meteorites, volcanic activity, floods, and climate alterations), via regular and irregular regional events, to anthropogenic and meanwhile more predictable events and processes, such as global warming. As a consequence of the mode of ‘life-in-all-places’, microbes are the closest, first-line contact with abiotic matter, the critical interface of the biosphere and geosphere–atmosphere, and link the physical and chemical Earth environment with life. Therefore, all such changes first impact the microbiosphere, which in turn influences nonmicrobial life.

There is also a physical and chemical molecular ecology determined by microbial action—habitat structuring and modification—which, on one hand, provides an additional dimension of dynamic change to the physical–chemical world in which life exists (Falkowski 2023), and, on the other, reacts to abiotic changes and provides a degree of self-regulation of the conditions of the biosphere and its interface with the geosphere–atmosphere (Gaia; Lovelock and Margulis 1974). Microbes repair ecosystem damage (for example by colonizing fresh lava following a volcanic eruption and creating the necessities of life for other organisms, see e.g. Dragone et al. 2023), rejuvenate habitats (for example, through biodegradation of toxic petroleum hydrocarbons following a spill at sea, e.g. Yakimov et al. 2007), and ‘buffer’ other organisms against change (for example, increases in temperature, e.g. Fontaine and Kohl 2023) or essential ecosystem services that assure biosphere stability, resilience and health (e.g. Ranheim Sveen et al. 2024).

## Anthropogenic activities perturb the microbiosphere and the services it provides

The natural regenerative capacity of microbes is huge but not infinite. Although the microbes in and around us mostly cannot be seen, and hence their health judged, the extraordinary nature of human beings has profound collateral impacts on the microbiosphere: the noise of liberty, wisdom, creativity, innovation, exploration–discovery, industry, resource extraction and waste production, trade, science, medicine—indeed any kind of ‘progress’—and of course policy relating to human activities, have the capacity to disturb the silent innocence and self-regulation of the pristine living world. This occurs because our irretrievable tendency to construct and enlarge our own human niche alters the Earth’s ecology and, as a consequence, other niches, all of which are colonized by microbes. Human activities that perturb the planet to an extent beyond the microbial repair capacity create ecological imbalances that reduce the health of the microbial component of the global metaorganism, its ecosystem services and role in maintaining the health of the planet, and thence the health of humanity. A current example is global warming, which reflects the anthropogenic production of carbon dioxide at a far greater rate than the capacity of microbes-chloroplasts to consume it, leading to climate change and climate tipping elements/points (critical thresholds beyond which a system reorganizes, such as the collapse of the West Antarctic and Greenland ice sheets—https://www.oecd.org/content/dam/oecd/en/publications/reports/2022/12/climate-tipping-points_9994de90/abc5a69e-en.pdf—see e.g. Lenton et al. 2008, Cavicchioli et al. 2019, Wunderling et al. 2024, and references therein). Other examples are to be found in the planetary boundaries (e.g. Richardson et al. 2023), all of which constitute serious challenges to human health.

Any significant disturbance of the microbiosphere constitutes a risk to health (e.g. https://www.woah.org/en/what-we-do/global-initiatives/one-health/). Microbial diseases of plants, animals, and humans are normal ecological interactions, battles, the outcome of which depends on the relative strengths of the combatants—often the underlying health of the hosts—the influence of their microbiota, and the transmission–colonization highways by which microorganisms propagate. Such highways frequently result from human behaviour (e.g. Baquero 2025, Timmis et al. 2025). Social inequality imposes disease vulnerability upon many deprived humans in our world, which are easily infected in poor-sanitation settings (e.g. Anand et al. 2023). But then the infecting microbes, having better adapted to humans in such settings, subsequently spread over the whole of humanity. The shift in the life expectancy pyramid in high-income countries towards older populations also offers a wealth of weak and disease-susceptible elderly hosts prone to infection. Travelling, including tourism and migration, facilitates microbial spread. Globalization in animal food production has reduced the diversity of food animals (https://news.un.org/en/story/2006/12/203402), a diversity that was once a barrier against pathogen propagation.

An excess of hygienic or therapeutic interventions (antimicrobials) in high-income countries, frequently based on a general culture of fear of all microbes (germophobia), including beneficial commensals, unwittingly creates empty niches that can be colonized by robust novel microbial invaders. The dominant psychological and cultural features and the resulting behaviour of humans (antimicrobial use, sanitation practices, pollution with microplastic particles, and so on) strongly influence the microbiosphere (Hernando-Amado et al. 2019). We know that there exists a potentially pathogenic microbiome in built environments, such as hospitals, public buildings, schools, public transport systems, and probably low-cost housing and, of course, farms. We also know that particles originating in sewage, soil, industrial facilities, mining, construction, or agricultural practices, among many others, can serve as meeting points for the coalescence of microbiotas of different origins, increasing interactions and the possible emergence of novel pathogens (Baquero et al. 2022).

Then there are the ‘time-space dimensions’ to the disturbance of the microbiosphere. Some microbes have been frozen in time for millennia or longer, for example in peatlands, hydrocarbon reservoirs, the cryosphere, and sedimentary geological formations, all of which can be released by human activities such as mining-resource extraction operations, and the thawing of cryosphere compartments like permafrost and glaciers due to human-caused global warming. Such old–new microbes, which have not participated in more recent global biosphere evolutionary processes, may have unanticipated consequences once they begin to interact with the biosphere. Other microbes may be geographically or otherwise separated from some members of the biosphere, and thus have not coevolved with them. Human activities that eliminate this separation, for example by invasion of particular, typically ‘wilderness’ or pristine habitats, in order to increase agricultural land area considered necessary to feed the growing human population, create new types of interactions, for example human exposure to new bat populations and the ‘new’ coronaviruses they carry, which may subsequently adapt to and infect human hosts. These issues, and others, inform why any human activity and interaction in nature can substantially affect a range of determinants of health, and testify to the need for inclusion of microbes in the concept of HiAP.

## The microbiosphere is the principal actor in One Health

The *One Health* concept (https://oneworldonehealth.wcs.org/About-Us/Mission/The-Manhattan-Principles.aspx; https://www.avma.org/sites/default/files/resources/onehealth_final.pdf; https://www.who.int/health-topics/one-health#tab=tab_1) recognizes that human health is inextricably intertwined with, and dependent upon, the health of other organisms of the biosphere and of the environment itself, and seeks to improve human health practices through holistic, integrated approaches that address determinants of health and risk factors in this wider context. From the foregoing, it is evident that the microbiosphere is a principal orchestrator of biosphere and environmental health, that is of One Health. One Health approaches therefore need to consider the influence and roles of microbiosphere health. However, our current understanding of key interactions between different environmental players that determine human health-relevant outcomes is limited. For example, simplistic views of the transfer of antimicrobial resistant pathogens from animals to humans are challenged by recent studies that suggest pathogenic strains circulating in humans, animals and the environment are distinct (Rousham et al. 2021, Thorpe et al. 2022)

## HiAP: the need to consider policy impacts on the microbiosphere

Health considerations of the microbiosphere generally focus on the effects of microbes, in particular the pathogens, on disease. However, since the microbiosphere is responsible for maintaining biosphere health, and hence human health, and is the first and most sensitive line of life challenged by human activities, it is essential to consider the effects of human activities on the microbiosphere. HiAP should therefore include an assessment of potential policy impacts on the health of the microbial world and their possible consequences for human health-related parameters in the broadest sense, including public health microbiology and the improvement of microbe–human-health education and behaviours.

Human activities and behaviours impacting microbial health are but one side of the coin: the other is that powerful microbial technologies have been developed that can, in appropriate situations, help restore and regenerate planetary and human health. These include *inter alia* vaccines, water purification and bioremediation technologies, and biopesticides. These can bring obvious benefits to health but could also be misused in ways that cause harm to health. An HiAP approach can help to identify these various impacts of microbial technologies. Thus, an understanding of microbial health can contribute to HiAP and HiAP can support positive outcomes from microbial technologies.

## Microbial Health, One Health, and HiAP: the need for education

There is an urgent need to correct prevalent misconceptions of many members of the lay public that microbes are necessarily harmful, and regularly evaluate progress by eliciting public attitudes (Jäckle and Timmis 2023, 2024). It is important to build trust and buy-in across society by refocusing narratives around the plentiful benefits of most microbes, to (re-)establish awareness for basic infection control behaviours against the ‘few’ bad microbes and, in turn, to normalize the relationship between humans and microbes, i.e. to counter germophobia. At the same time, it is essential to explain the vital role of the microbiosphere in biosphere and human heath, its universal connectivity, and its vulnerability to perturbation by human activities. This education is needed at all levels of society and all ages, but particularly at school, for example as is being developed by the International Microbiology Literacy Initiative (Timmis et al. 2019, 2024, van Beek et al. 2024).

## The need for new research focused on Microbial Health, One Health, and HiAP

Achievement of the goals of HiAP through due consideration of impacts of policies on the microbiosphere and their potential knock-on effects on biosphere and human health, will necessitate the allocation of significant resources. We need new research because we lack methods and protocols to measure the precise impact of human policies on our life-sustaining microbial world. Even less is known about effective systems for microbial restoration after ecological damage, and the precise health risks of alterations in the microbiosphere are hardly known. Such knowledge deficiencies should be addressed urgently by developing research focusing on policy areas with a scientific epicenter in microbiology research. Whole World institutions and scientific societies, under the coordination and governance of international health organizations, such as the WHO, should be able to promote local, national, or international research tools in a landscape of intersectoral planning to ensure the maintenance of a healthy equilibrium between human development and the preservation of the microbiosphere.

Recommendations:

1. Microbial Health and One Health should be integrated into HiAP work, and involve experts in microbial health and technologies.
2. Training programmes in Microbial Health, One Health, and HiAP should be developed worldwide to satisfy the need for such experts. There needs to be coordination support for a network of trainers and adaptations of training materials for different sectors (e.g. health, environment, and housing).
3. Training resources and tools, and capacity-building opportunities, need to be made available.
4. Existing experts should be recruited to provide advice through the Global Network for HiAP.
5. Standards need to be established for transforming education for health workforce towards social determinants of health.
6. Resources for systematically and actively improving, and properly communicating, knowledge on ‘healthy’ microbe–human interactions to normalize behaviours should be made available.
7. Through improved intersectoral planning, for health and health equity, including impacts on microbial health, links need to be established between WHO governance and programme tools.
8. Government policy-makers, programme leaders, and health provider groups need to be supported to improve intersectoral action and coherence in policies, services, and programmes responding to disadvantaged groups’ needs. The role of microbial health in the health impact assessment of such vulnerable groups should be further investigated.
9. Education programmes need to be developed and rolled out that create an understanding of the microbiosphere and its central importance in the health of humans, the biosphere and the environment.

## References

[bib1] Anand S, Hallsworth JE, Timmis J et al. Weaponising microbes for peace. Microb Biotechnol. 2023;16:1091–111. 10.1111/1751-7915.14224. 36880421 PMC10221547

[bib2] Baquero F, Coque TM, Guerra-Pinto N. et al. The influence of coalescent microbiotic particles from water and soil on the evolution and spread of antimicrobial resistance. Front Environ Sci. 2022;10:824963.

[bib3] Baquero F . Neglected human risk factors determining sustainable health by microbial causes: individual versus social conducts, scientific versus stultified behaviour. Microb Biotechnol. 2025;18:e70097. 10.1111/1751-7915.70097. 39969676 PMC11837811

[bib4] Bar-On YM, Phillips R, Milo R. The biomass distribution on Earth. Proc Natl Acad Sci USA. 2018;115:6506–11. 10.1073/pnas.1711842115. 29784790 PMC6016768

[bib5] Bertrand JC, Brochier-Armanet C, Gouy M. et al. 2015. For three billion years, microorganisms were the only inhabitants of the Earth. In: Bertrand JC, Caumette P, Lebaron P, Matheron R, Normand P, Sime-Ngando T. (eds) Environmental Microbiology: Fundamentals and Applications. Dordrecht: Springer. 10.1007/978-94-017-9118-2_4.

[bib6] Cavicchioli R, Ripple WJ, Timmis KN. et al. Scientists’ warning to humanity: microorganisms and climate change. Nat Rev Microbiol. 2019;17:569–86. 31213707 10.1038/s41579-019-0222-5 PMC7136171

[bib7] Dragone NB, Whittaker K, Lord OM et al. The early microbial colonizers of a short-lived volcanic island in the Kingdom of Tonga. mBio. 2023;14:e03313–22. 10.1128/mbio.03313-22. 36629429 PMC9972954

[bib8] Falkowski PG, Fenchel T, Delong EF. The microbial engines that drive Earth’s biogeochemical cycles. Science. 2008;320:1034–9. 10.1126/science.1153213. 18497287

[bib9] Falkowski PG. Life’s Engines: How Microbes Made Earth Habitable. Princeton, NJ: Princeton University Press, 2023. 10.2307/j.ctv345pcx3.

[bib10] Fontaine SS, Kohl KD. The microbiome buffers tadpole hosts from heat stress: a hologenomic approach to understand host-microbe interactions under warming. J Exp Biol. 2023;226:jeb245191. 10.1242/jeb.245191. 36546449 PMC10086385

[bib11] Gumerov VM, Andrianova EP, Matilla MA et al. Amino acid sensor conserved from bacteria to humans. Proc Natl Acad Sci USA. 2022;119:e2110415119. 10.1073/pnas.2110415119. 35238638 PMC8915833

[bib12] Hernando-Amado S, Coque TM, Baquero F. et al. Defining and combating antibiotic resistance from One Health and Global Health perspectives. Nat Microbiol. 2019;4:1432–42. 31439928 10.1038/s41564-019-0503-9

[bib14] Jäckle S, Timmis JK. Esoteric beliefs and CAM impact SARS-CoV-2 immunization drivers, uptake and pediatric immunization views in Germany. Npj Vaccines. 2024;9:137. 10.1038/s41541-024-00928-7. 39097580 PMC11297982

[bib13] Jäckle S, Timmis JK. Left–right-position, party affiliation and regional differences explain low COVID-19 vaccination rates in Germany. Microb Biotechnol. 2023;16:662–77. 10.1111/1751-7915.14210. 36622064 PMC9948222

[bib15] Knoll A. Life on a Young Planet. Princeton, NJ: Princeton University Press, 2015. 10.1515/9781400866045.

[bib16] Lenton TM, Held H, Kriegler E. et al. Tipping elements in the Earth’s climate system. Proc Nat Acad Sci USA. 2008;105:1786–93. 10.1073/pnas.0705414105. 18258748 PMC2538841

[bib17] Locey KJ, Lennon JT. Scaling laws predict global microbial diversity. Proc Natl Acad Sci. 2016;113:5970–5. 10.1073/pnas.152129111. 27140646 PMC4889364

[bib18] Lovelock JE, Margulis L. “Atmospheric homeostasis by and for the biosphere: the gaia hypothesis”. Tellus A. 1974;26:2–10. 10.3402/tellusa.v26i1-2.9731.

[bib19] Matilla MA, Genova R, Martín-Mora D et al. The cellular abundance of chemoreceptors, chemosensory signaling proteins, sensor histidine kinases, and solute binding proteins of *Pseudomonas aeruginosa* provides insight into sensory preferences and signaling mechanisms. Int J Mol Sci. 2023a;24:1363. 10.3390/ijms24021363. 36674894 PMC9864684

[bib20] Matilla MA, Monteagudo-Cascales E, Krell T. Advances in the identification of signals and novel sensing mechanisms for signal transduction systems. Environ Microbiol. 2023b;25:79–86. 10.1111/1462-2920.16142. 35896893

[bib21] McCutcheon JP . From microbiology to cell biology: when an intracellular bacterium becomes part of its host cell. Curr Opin Cell Biol. 2016;41:132–6. 10.1016/j.ceb.2016.05.008. 27267617 PMC5772596

[bib22] MohammadiPeyhani H, Hafner J, Sveshnikova A. et al. Expanding biochemical knowledge and illuminating metabolic dark matter with ATLASx. Nat Commun. 2022;13:1560. 10.1038/s41467-022-29238-z. 35322036 PMC8943196

[bib23] Moore EK, Jelen BI, Giovannelli D. et al. Metal availability and the expanding network of microbial metabolisms in the Archaean eon. Nat Geosci. 2017;10:629–36.

[bib24] Ranheim Sveen T, Hannula SE, Bahram M. Microbial regulation of feedbacks to ecosystem change. Trends Microbiol. 2024;32:68–78. 37500365 10.1016/j.tim.2023.06.006

[bib25] Richardson K. et al. Earth beyond six of nine planetary boundaries. Sci Adv. 2023;9. 10.1126/sciadv.adh2458. PMC10499318 37703365

[bib26] Rousham EK, Asaduzzaman M, Mozmader TIMAU et al. Human colonization with extended-spectrum beta-lactamase-producing *E. coli* in relation to animal and environmental exposures in Bangladesh: an observational One Health study. Environ Health Perspect. 2021;129:37001. 10.1289/EHP7670. 33656920 PMC7929562

[bib27] Thorpe HA, Booton R, Kallonen T et al. A large-scale genomic snapshot of *Klebsiella* spp. isolates in Northern Italy reveals limited transmission between clinical and non-clinical settings. Nat Microbiol. 2022;7:2054–67. 10.1038/s41564-022-01263-0. 36411354 PMC9712112

[bib28] Timmis K, Cavicchioli R, García JL et al. The urgent need for microbiology literacy in society. Environ Microbiol. 2019;21:1513–28. 30912268 10.1111/1462-2920.14611

[bib29] Timmis K, Hallsworth JE, McGenity TJ. et al. A concept for international societally relevant microbiology education and microbiology knowledge promulgation in society. Microb Biotechnol. 2024;17:e14456. 10.1111/1751-7915.14456. 38801001 PMC11129164

[bib30] Timmis K, Karahan ZC, Ramos JL et al. Microbes saving lives and reducing suffering. Microb Biotechnol. 2025;18:e70068. 10.1111/1751-7915.70068. 39844583 PMC11754571

[bib31] Van Beek R, Spijkerman DJC, van der Burgt N. et al. Guidelines to support the design, and selection and appraisal of multimedia teaching aids for microbiology education. Microb Biotechnol. 2024;17:e14553. 10.1111/1751-7915.14553. 39163108 PMC11334907

[bib34_296_085725] Wunderling N., von der Heydt, Aksenov Y.“Climate Tipping Point Interactions and Cascades: A Review Earth Syst 15:.” Dynamis. 2024. 15:41–74. 10.5194/esd-15-41-2024.

[bib32] Yakimov MM, Timmis KN, Golyshin PN. Obligate oil-degrading marine bacteria. Curr Opin Biotechnol. 2007;18:257–66. 10.1016/j.copbio.2007.04.006. 17493798

[bib33] Zhang XH, Anderson KM, Dong HM. et al. The cell-type underpinnings of the human functional cortical connectome. Nat Neurosci. 2024;28:150–60. 10.1038/s41593-024-01812-2. 39572742 PMC12903763

